# Usefulness of intraoperative transesophageal echocardiography for hemodynamic management of liver transplantation in a patient with massive polycystic liver disease: a case report

**DOI:** 10.1186/s40981-023-00646-4

**Published:** 2023-08-26

**Authors:** Yudai Ueda, Takahiro Kawaji, Hidefumi Komura, Yoshitaka Hara, Naohide Kuriyama, Tomoyuki Nakamura, Osamu Nishida

**Affiliations:** Department of Anesthesiology and Critical Care Medicine, Fujita Health University School of Medicine, 1-98 Dengakugakubo, Kutsukake-Cho, Toyoake, Aichi 470-1192 Japan

**Keywords:** Liver transplantation, Polycystic liver disease, Transesophageal echocardiography

## Abstract

**Background:**

Hemodynamic management during anesthesia in liver transplantation for patients with polycystic liver disease (PLD) can be more challenging because of the bleeding and hemodynamic alterations due to the markedly enlarged liver. We hereby report a case of PLD wherein transesophageal echocardiography (TEE) was employed for optimal hemodynamic monitoring during liver transplantation.

**Case presentation:**

A 61-year-old man was scheduled to undergo liver transplantation for massive PLD. Hemodynamic instability was associated with mechanical displacement of the giant cystic liver. TEE results revealed the collapse of the inferior vena cava due to liver displacement. TEE also detected intrathoracic hemorrhage triggered by detachment from the markedly enlarged liver.

**Conclusion:**

TEE is a valuable monitoring tool for sharing information with surgeons and diagnostic modality for finding the source of bleeding in liver transplantation for PLD and may contribute majorly to the quality of perioperative management.

## Background

In severe cases of polycystic liver disease (PLD), numerous cysts develop within the liver causing mass effects such as abdominal distension, dyspnea, and severe liver damage [1]. Percutaneous therapies such as cyst aspiration with sclerotherapy and transcatheter hepatic arterial embolization have certain limitations, and liver transplantation is currently the only cure for PLD [2]. Hemodynamic instability usually occurs during liver transplantation due to hemorrhage, fluid shifts caused by reperfusion, and reperfusion syndrome [3, 4]. Hemodynamic management is more challenging in patients with PLD because of the bleeding associated with extensive dissection due to the markedly enlarged liver and hemodynamic alterations caused by the displacement of the cystic liver. Transesophageal echocardiography (TEE) is an effective circulatory monitor that is widely used not only in cardiac surgery but also in noncardiac surgery that provides a variety of useful intraoperative information [5]. Herein, we report a case in which TEE proved to be useful in managing the blood circulation during liver transplantation for massive PLD.

### Case presentation

**Fig. 1:**
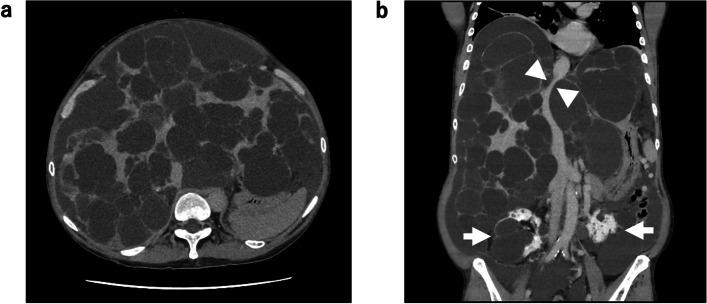
Abdominal contrast-enhanced computed tomography images △Narrowed inferior vena cava (IVC) due to compression by surrounding cysts.  → Polycystic kidneys

A 61-year-old man (height, 173 cm; weight, 75 kg) was diagnosed with PLD more than 10 years prior. Cyst aspiration with sclerotherapy and hepatic arterial embolization was repeatedly performed with limited therapeutic effects. Owing to abdominal distention, worsening pain, and liver function tests revealing abnormalities, he was scheduled for liver transplantation of the right hepatic lobe from his wife. Preoperative computed tomography revealed a giant cystic liver occupying the abdominal cavity as well as an umbilical hernia (Fig. 1). The inferior vena cava (IVC) was narrowed due to compression by the surrounding cysts. Although the patient had polycystic kidneys, his renal function test results were within the normal range. His preoperative blood coagulation tests results were normal. Furthermore, transthoracic echocardiography revealed sound ventricular contractility, and esophagogastroduodenoscopy showed the absence of gastroesophageal varices.

The patient underwent intraoperative electrocardiography, peripheral oxygen saturation measurement, and invasive arterial pressure monitoring. General anesthesia was induced with 5 mg of midazolam, 0.2 μg/kg/min of remifentanil, and 70 mg of rocuronium using rapid sequence induction and intubation technique. Moreover, the inserted TEE probe revealed a narrowed IVC compressed by numerous cysts. However, the intracardiac volume was maintained. Triple-lumen central venous catheters for drug administration and pulmonary artery catheters for hemodynamic monitoring were placed via the right internal jugular vein. The tip of the pulmonary artery catheter was placed in the main trunk of the pulmonary artery under fluoroscopic guidance. Additionally, a 12-Fr sheath introducer was placed in the left internal jugular vein. General anesthesia was maintained with 2% sevoflurane, 0.15 μg/kg/min remifentanil, and 7 μg/kg/min rocuronium.

**Fig. 2:**
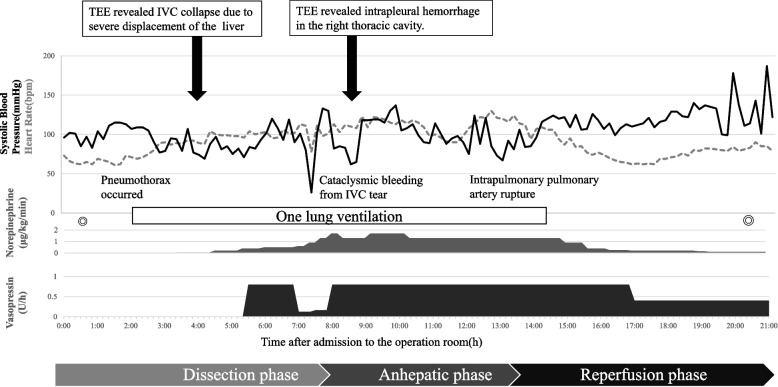
Intraoperative hemodynamics and events. Transesophageal echocardiography (TEE) indicated inferior vena cava (IVC) collapse due to severe mechanical liver displacement. Since the IVC injury could result in massive bleeding, the circulation was maintained with a rapid transfusion while observing the TEE. TEE also detected fluid accumulation in the right thoracic cavity. We suspected an injury to the lung and the pulmonary artery, following which hemostasis and repairment were performed sBP, systolic blood pressure; HR, heart rate; ◎, start and end of the operation

**Fig. 3:**
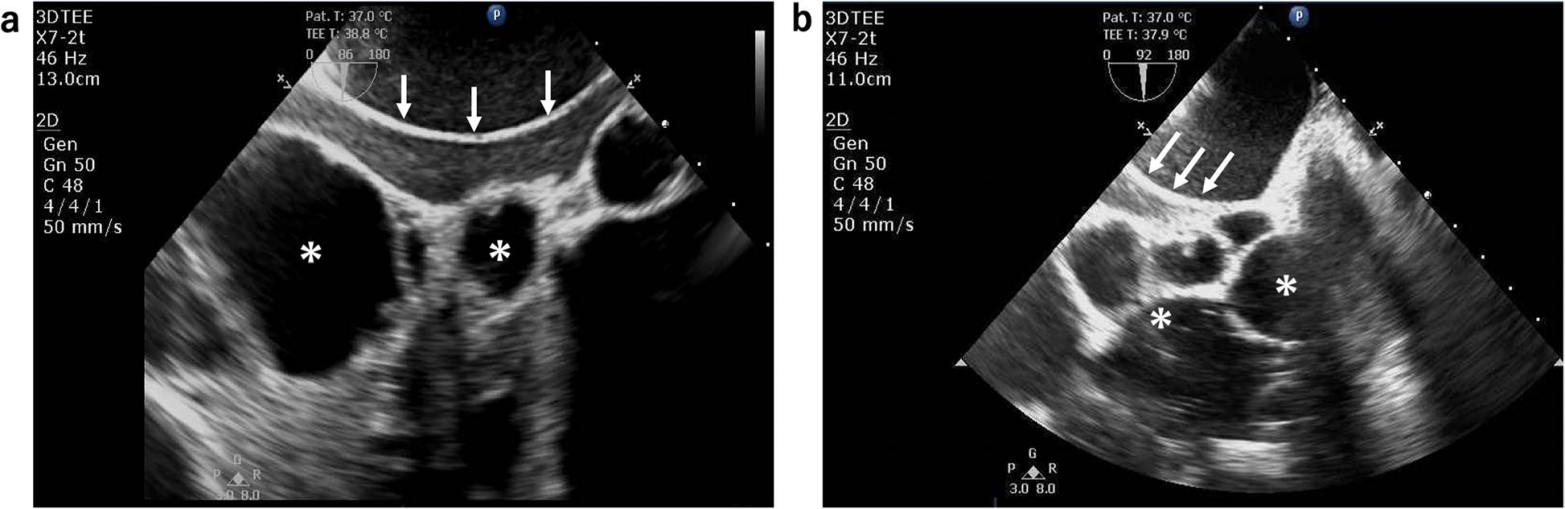
Transesophageal echocardiography (TEE) images of the intrahepatic inferior vena cava (IVC). Before the mechanical liver displacement, the IVC (white arrows) was surrounded by various cysts (asterisks), but was not collapsed (**a**). TEE indicated IVC collapse (**b**) due to severe mechanical liver displacement

Intraoperative events with hemodynamics are shown in Fig. 2. During the dissection phase, as many cysts as possible were evacuated to decrease the liver size while detaching the liver from the surrounding tissue. Pneumothorax occurred when the adhesion between the liver and diaphragm was dissected, and one-lung ventilation with a bronchial blocker was initiated. Hemodynamic instability associated with mechanical displacement of giant polycystic liver and IVC collapse due to severe liver displacement were detected by TEE (Fig. 3). These findings were shared with the surgeon, and the venous return was increased to maintain the circulation. Furthermore, access to the IVC led to venous tearing and cataclysmic bleeding during dissection because the border between the IVC and cyst was unclear. Accordingly, blood products were adequately transfused while carefully monitoring the blood pressure, central venous pressure, pulmonary artery pressure, and TEE. The surgeon clamped the suprahepatic and subhepatic IVCs and performed a total hepatectomy.

During the anhepatic phase, hemodynamic instability persisted, but there was no active bleeding in the abdominal cavity. TEE, which was employed to identify potential sources of bleeding, revealed pleural effusion suggestive of intrapleural hemorrhage in the right thoracic cavity, and lung injury and hematoma were confirmed. A partial pneumonectomy was immediately performed; however, the intrathoracic hemorrhage persisted after graft reperfusion, and the patient’s hemodynamics remained unstable. Therefore, the surgeon confirmed intrapulmonary artery rupture via a semi-clamshell incision approach and repaired the pulmonary artery tears. Thereafter, the patient’s hemodynamics stabilized, and surgery was completed. The durations of surgery and anesthesia were 1161 and 1282 min, respectively. Blood loss and urine output were 34,098 g and 6100 mL, respectively. The patient was administered 12,500 mL of crystalloid, 2750 mL of 5% albumin, 15,680 mL of packed red blood cells, 26,880 mL of fresh-frozen plasma, 150 mL of cryoprecipitate, and 1400 mL of platelet concentrate.

The patient was transferred to the intensive care unit (ICU), intubated, and sedated with 0.05 mg/kg/h midazolam. On postoperative day 4, hepatic artery thrombectomy was performed. The patient was weaned off the ventilator on postoperative day 6. The transplanted liver functioned satisfactorily, and the patient was discharged from the ICU on postoperative day 12. On postoperative day 90, the patient was discharged without any signs of liver transplant rejection.

## Discussion

Hemodynamic management is essential in anesthesia during liver transplantation in patients with PLD owing to bleeding complications in the cyst induced by the enlarged liver and may result from dissection and displacement. Enlarged polycystic livers complicate dissection, whereas typical cirrhotic livers are small and can be easily manipulated [6]. In addition, because patients with PLD commonly undergo cyst aspiration and hepatic artery embolization with sclerotherapy before transplantation, a large cystic liver is often tightly adherent to the surrounding tissue. Therefore, many blood vessels, particularly the IVC, are often displaced or compressed, and tearing or rupture can result in excessive bleeding. Perioperative mortality from liver transplantation for PLD is higher than that for other diagnoses, owing to the complexity of the surgical procedure [7].

Intraoperative TEE is a valuable monitoring and diagnostic modality for liver transplantation in PLD. Pulmonary artery catheters and continuous cardiac output monitoring devices are commonly used to monitor hemodynamics during liver transplantation. However, TEE can provide additional information otherwise difficult to detect. TEE during liver transplantation can not only detect dynamic left ventricular outflow obstruction and intracardiac air embolism but also provide extracardiac information such as IVC stenosis [8]. In our case, TEE revealed that hemodynamic instability during the dissection phase was due to IVC collapse associated with mechanically enlarged polycystic liver displacement. Therefore, we shared the findings with the surgeon, and to maintain cardiac output, the venous return was increased. Because PLD is often complicated by polycystic kidney disease, maintaining venous perfusion may be renoprotective in terms of avoiding renal congestion. Aubuchon et al. reported the detection of thrombotic stenosis of the IVC during liver transplantation in PLD [8], but there is no report that TEE detected IVC collapse due to mechanical displacement of the giant cystic liver. In this case, TEE was also useful for the early detection of right intrathoracic hemorrhage. While monitoring circulation, a rapid blood transfusion was performed in case of massive bleeding during the dissection of the adhesion between the IVC, diaphragm, and lungs. If hemostasis is difficult, circulatory support via a veno-venous bypass may be necessary. In the reperfusion phase, TEE helps detect intrathoracic hemorrhage as the cause of prolonged circulatory failure and provides useful intraoperative diagnostic information. In this case, the cause of intrathoracic hemorrhage was the tearing of the pulmonary artery. The cyst was strongly adherent to the right diaphragm and tore the lung and pulmonary artery during dissection. Adhesions may have extended beyond the diaphragm as a result of repeated cyst aspiration with sclerotherapy. Therefore, the spread of inflammation around the cyst and perforation of the cyst were considered when approaching the liver cysts at the perimeter of the diaphragm. The pulmonary artery injury was not considered to be a complication of the pulmonary artery catheter because its tip was placed in the main trunk of the pulmonary artery under fluoroscopic guidance. TEE revealed that its position did not change during the surgery. Regarding the surgical approach for liver transplantation in patients with PLD, Le Roy et al. reported a modified total hepatectomy that limits hazardous liver manipulation and improves exposure of the IVC [7]. Left lateral sectionectomy post dissection of hepatic pedicle followed by total hepatectomy preserves the IVC and its blood flow, which could have prevented intraoperative complications in the present case. Therefore, anesthesiologists should recognize the aforementioned alternative surgical approach and suggest it to the surgical team preoperatively.

Current guidelines consider TEE a relative contraindication for patients with esophageal varices [9]. However, post-TEE gastrointestinal bleeding in patients with esophageal varices was not significantly higher than that in patients without varices [10]. Major complications due to TEE occurred in only 0.47% of the 2706 liver transplant recipients, with no significant change in the risk of bleeding due to gastric or esophageal varices [11]. Furthermore, owing to the symptoms of mass effect, patients with PLD are significantly more likely to undergo liver transplantation than those with portal hypertension [12]. Therefore, liver transplantation for PLD is unlikely to be complicated by esophageal varices and may be a good indication for TEE usage.

Circulatory management is more challenging in cases of PLD because of the risk of hemorrhage associated with extensive detachment due to a significantly enlarged liver and hemodynamic alterations caused by displacement of the organ. TEE may contribute to the quality of perioperative management of liver transplantation for PLD, not only as a circulatory monitor but also by providing essential intraoperative diagnostic information.

## Data Availability

Not applicable.

## Abbreviations

IVC: Inferior vena cava
ICU: Intensive care unit
PLD: Polycystic liver disease
TEE: Transesophageal echocardiography
